# Prenatal Exposure to Glycol Ethers and Neurocognitive Abilities in 6-Year-Old Children: The PELAGIE Cohort Study

**DOI:** 10.1289/EHP39

**Published:** 2016-10-14

**Authors:** Rémi Béranger, Ronan Garlantézec, Gaïd Le Maner-Idrissi, Agnès Lacroix, Florence Rouget, Jessica Trowbridge, Charline Warembourg, Christine Monfort, Florent Le Gléau, Marylène Jourdin, Luc Multigner, Sylvaine Cordier, Cécile Chevrier

**Affiliations:** 1 INSERM (Institut national de la santé et de la recherche médicale) U1085-IRSET, University Rennes 1, Rennes, France; 2 University Hospital Rennes, Rennes, France; 3 Research Centre for Psychology, Cognition and Communication (CRPCC EA 1285), University of Rennes 2, Rennes, France; 4 Department of Pediatrics, Rennes University Hospital, Rennes, France; 5 LABOCEA (Laboratoire public Conseil, Expertise et Analyse), Plouzané, France

## Abstract

**Background:**

Glycol ethers (GE) are widely used organic solvents. Despite the potential neurotoxicity of several families of organic solvents, little is known about the impact of GE on the neurodevelopment of infants and children.

**Objectives:**

We investigated the relation between urinary concentrations of GE metabolites in pregnant women and neurocognitive abilities in their 6-year-old children in the PELAGIE mother–child cohort.

**Methods:**

Five GE metabolites were measured in first morning void urine samples of 204 French pregnant women in early pregnancy (< 19 weeks of gestation). Psychologists assessed the neurocognitive abilities of their 6-year-old children with the Wechsler Intelligence Scale for Children IV (WISC) and the Developmental Neuropsychological Assessment (NEPSY). We analyzed the results with linear (WISC) and Poisson regression models (NEPSY), adjusted for potential confounders, including child’s stimulation at home.

**Results:**

GE metabolites were detected in 90–100% of maternal urine samples. The WISC Verbal Comprehension score was significantly lower for children with the highest tertile of urinary phenoxyacetic acid (PhAA) [β (third vs. first tertile) = –6.53; 95% CI: –11.44, –1.62]. Similarly, the NEPSY Design Copying subtest score was lower in those with the highest tertile of urinary ethoxyacetic acid (EAA) [β (third vs. first tertile) = –0.11; 95% CI: –0.21, 0.00]. The other GE metabolites we studied were not significantly associated with WISC or NEPSY scores.

**Conclusions:**

Prenatal urine concentrations of two GE metabolites were associated with lower WISC Verbal Comprehension Index scores and NEPSY Design Copying subscale scores, respectively, at age 6 years. PhAA is the primary metabolite of 2-phenoxyethanol (EGPhE), which is commonly found in cosmetics, and precursors of EAA are frequently used in cleaning agents. Additional research is needed to confirm our findings and further explore potential effects of prenatal GE exposures on neurocognitive performance in children.

**Citation:**

Béranger R, Garlantézec R, Le Maner-Idrissi G, Lacroix A, Rouget F, Trowbridge J, Warembourg C, Monfort C, Le Gléau F, Jourdin M, Multigner L, Cordier S, Chevrier C. 2017. Prenatal exposure to glycol ethers and neurocognitive abilities in 6-year-old children: the PELAGIE cohort study. Environ Health Perspect 125:684–690; http://dx.doi.org/10.1289/EHP39

## Introduction

Glycol ethers (GE) are oxygenated solvents that are highly miscible in water and oils. Their low acute toxicity has favored their inclusion in a large number of products used occupationally and domestically, including water-based paints, cleaning products, liquid soaps, cosmetics, and even some pharmaceutical products (Table 1).

**Table 1:** Main parent compounds for each alkyloacetic metabolite and their sources in France (2000–2006).

Metabolites	Parent glycol ethers	Cosmetics	Cleaning agents	Paint, varnishes, and inks	Biocides	Drugs
MAA	DEGME, TEGME, TEDGME	Not reported	Yes	Not reported	Not reported	Not reported
EAA	DEGEE, TEGEE	Yes	Yes^*a*^	Yes	Yes	Yes
EEAA	DEGEE, TEGEE	Yes	Yes^*a*^	Yes	Yes	Yes
BAA	EGBE, DEGBE	Yes	Yes^*a*^	Yes^*a*^	Yes	Not reported
PhAA	EGPhE	Yes^*a*^	Not reported	Not reported	Yes	Yes
Abbreviations: BAA, 2-butoxyacetic acid; DEGBE, 2-(2-butoxyethoxy)ethanol; DEGEE, 2-(2-ethoxyethoxy)ethanol; DEGME, 2-(2-methoxyethoxy)ethanol; EAA, ethoxyacetic acid; EEAA, ethoxyethoxyacetic acid; EGBE, 2-butoxyethanol; EGPhE, 2-phenoxyethanol; MAA, methoxyacetic acid; PhAA, phenoxyacetic acid; TEGDME, 1,2-bis(methoxyethoxy)ethane; TEGEE, 2-[2-(2-ethoxyethoxy)ethoxy]ethanol; TEGME, 2-[2-(2-methoxyethoxy)ethoxy]ethanol. Data from AFSSET (2008). ^***a***^Considered as widely used in these products, according to a collective expertise of the French Agency for Food, Environmental and Occupational Health and Safety (AFSSET 2008).						

Annual usage of GE was estimated at 350,000 tons in Europe in 2006 (AFSSET 2008) and is similar in the United States (OSPA 2016). The general population has had regular, frequent contact with GE solvents since the 1960s (INSERM 1999). Studies in France and Germany of urinary biomarkers of GE exposure have shown that the majority of the general population, including pregnant women, is exposed to GE (Ben-Brik et al. 2004; Fromme et al. 2013; Garlantézec et al. 2012).

Exposure to GE occurs primarily via inhalation and dermal routes. GE with primary hydroxy groups in the molecule, such as those derived from ethylene GE, are readily metabolized by aldehyde and alcohol dehydrogenase to acid metabolites, their most toxicologically significant metabolic products, and excreted in urine (Johanson 1996). Experimental and epidemiological studies have documented evidence of reproductive and hematologic effects of some ethylene GE (INSERM 1999, 2006). Moreover, experimental studies have reported behavioral impairment and neurochemical alteration of the central nervous system in young rats after maternal exposure to 2-methoxyethanol (EGME) and 2-ethoxyethanol (EGEE) (Nelson and Brightwell 1984; Nelson et al. 1984).

To our knowledge, no human studies have explored the potential consequences of GE exposure during pregnancy on the neurodevelopment of infants/children, and only a few have examined the effect of prenatal exposure to organic solvents in general on human neurodevelopment. These studies reported that maternal occupational exposure during pregnancy was associated with lower scores for visuospatial and graphomotor ability (Till et al. 2001a), visual ability (Till et al. 2001b, 2005), and neurobehavioral performance (Laslo-Baker et al. 2004; Pelé et al. 2013).

Our prospective study design enabled us to investigate the effect of prenatal exposure to GE on the neurocognitive abilities of children, by using maternal urine samples collected in early pregnancy and standardized neuropsychological tests performed when the children reached 6 years of age.

## Methods

### Population

The PELAGIE (*Perturbateurs endocriniens, Étude Longitudinale sur les Anomalies de la Grossesse, l’Infertilité et l’Enfance*) mother–child cohort included 3,421 pregnant women from Brittany, France, between 2002 and 2006. Women were recruited before the 19th week of gestation [median, 10 weeks; interquartile range (IQR), 8–11; minimum–maximum, 4–17] by their gynecologist, obstetrician, or ultrasonographer at the first visit of the pregnancy (Garlantézec et al. 2009; Petit et al. 2012). A subcohort of 591 mother–child pairs was randomly selected from the live-born singleton children of study participants for a neurobehavioral assessment at age 6 years. Twenty were excluded for preterm birth before 35 weeks’ gestation, neonatal respiratory distress or hospitalization, or Down syndrome; 125 could not be reached by telephone; and 18 more were excluded because the child had previously undergone neuropsychological or behavioral tests (to avoid bias due to the learning effect). Among the 428 remaining families, 287 (67%) mothers agreed to participate with their child in the neuropsychological follow-up. We had sufficient remaining urine from the initial samples to measure GE metabolites for 204 (71%); the samples for the other women had been entirely used up for other urine assays (Cartier et al. 2016). This subpopulation has socioeconomic and maternal behavioral characteristics very similar to those of the rest of the initial population of the PELAGIE cohort (see Table S1).

### Data Collection

At inclusion, women completed a self-administered questionnaire about their family, social and demographic characteristics, diet, and lifestyle. When the children were 2 and 6 years old, mothers completed additional self-administered questionnaires, providing longitudinal information on sociodemographic characteristics, lifestyle factors, and the children’s health as well as their environmental exposures. To assess exposure to domestic solvents during childhood, we asked about the frequency of cleaning product use as well as of domestic use of products likely to contain solvents for house renovation and for hobbies.

### Neuropsychological Assessment

Two psychologists blinded to exposure status visited all families. One performed the children’s neuropsychological assessments, while the other interviewed the mother, tested her cognitive performance, and assessed the home environment. These psychologists were supervised by four pediatric neuropsychologists in meetings held every 2 months.

The Wechsler Intelligence Scale for Children, 4th edition (WISC-IV), was used to assess the children’s neurocognitive abilities (Wechsler 2003). Psychologists generated scores for two domains: the Verbal Comprehension Index (WISC-VCI; three subtests: Similarities, Vocabulary, and Comprehension), which measures verbal concept formation and is a good predictor of school readiness, and the Working Memory Index (WISC-WMI; two subtests: Digit Span and Letter-Number Sequencing), which assesses children’s ability to memorize new information, their short-term memory, and their ability to concentrate and manipulate information. Scores are based on a scale with a mean of 100 and a standard deviation of 15 (minimum–maximum, 40–160). Higher scores indicate better neurocognitive abilities. Some values were missing (WISC-VCI = 4, WISC-WMI = 12), mainly for subtests at the end of the session. Children’s visuospatial ability was assessed with two subtests of the Developmental Neuropsychological Assessment (NEPSY) battery (Design Copying and Arrows) (Korkman et al. 1998), similarly to the study by Till et al. (2001a). For each subtest, the mean scaled score was 10 and the standard deviation was 3 (minimum-maximum, 1–19). Higher scores indicate better neuropsychological functioning.

We used the Wechsler Adult Intelligence Scale, 3rd revision (WAIS-III) (Wechsler 1997) to estimate the mothers’ Verbal Intelligence Quotients and assess their general knowledge, language, reasoning, and memory skills. The evaluation of the quality and extent of stimulation available to the child in the home environment used the HOME inventory (Home Observation for Measurement of the Environment) (Caldwell and Bradley 1979), with a higher HOME score indicating a more supportive and stimulating home environment.

***Chemical analyses.*** At inclusion, each woman mailed a first morning void urine sample in a 10 mL test tube (95 × 16-mm polypropylene, with wing plug; nitric acid was used as conservative) to our laboratory. Samples were sent in an opaque and rigid box at ambient temperature. Upon arrival, urine samples were frozen at –20°C until analysis. The median time from urine sample collection by a participant to receipt of the sample by the study laboratory was 2 days (IQR, 1–3; minimum–maximum, 0–48), and the median duration of storage 100 months (IQR, 95–110; minimum–maximum, 91–114). In a previous study based on the PELAGIE cohort (Cordier et al. 2012), the influence of transportation time at room temperature (1, 2–3, 4–34 days; *n* = 246, 207, and 124, respectively) and of the duration of storage at –20°C (< 865, 865–1,095, 1,096–1,375, ≥ 1,376 days; *n* = 145, 145, 145, and 145, respectively) showed negligible effect on the five urinary GE metabolite concentrations and detection frequencies.

One milliliter of dichloromethane was used to extract five urinary GE metabolites in 2-mL urinary samples simultaneously (Carlo Erba, Cornaredo, Italy). Derivatization was performed with pentafluorobenzyl bromide as the alkylation reagent (Aldrich, St. Louis, MO, USA) in 5 mL tetrabutylammonium hydrogen sulfate buffer (Molekula, Lyon, France) at pH 4.5. Butoxy-D9 acetic acid (Dr Ehrenstorfer, Augsburg, Germany) was added as an internal standard. The vials were shaken for 16 hr at 37°C. We extracted the organic phase and used a Turbovap II (Zymarck, Hopkinton, MA, USA) to evaporate it to 0.25 mL. The analysis was conducted with gas chromatography (HP 7890A; Agilent, Santa Clara, CA, USA) and triple quadrupole mass spectrometry (HP 7000C; Agilent), with a Varian Factor Four VF-1ms capillary column (15 m × 0.25 mm, 0.1 μm; Agilent). We used one transition for quantification and two for qualification. Concentration range linearity was observed from 0.01 to 1.00 mg/L for the urinary GE metabolites. The average recovery was 99–104%, and the coefficients of variation ranged from 15% to 20%. The limit of detection (LOD) was 0.003 mg/L for all metabolites.

Exposure to lead, which was evaluated as a potential confounder, was measured as acid-leachable lead in living room floor dust (standard protocol for dust sampling: ASTM E1792-03–Standard Specification for Wipe Sampling Materials for Lead in Surface Dust). Procedures for analyses are presented elsewhere (Le Bot et al. 2011).

***Data analyses***. Values below the LOD for GE urinary metabolites were randomly imputed from a log-normal probability distribution derived by a maximum-likelihood estimation method (Jin et al. 2011). Overall, the imputed value accounted for 0–10% of the values for the different metabolites (Table 2). WISC-VCI scores were imputed for 4 children with missing data on one of the three WISC-VCI subtests by using linear regression models of the data for the subtests with known values. We used the same approach to impute WISC-WMI scores for 8 children with missing data for one or two of the two subtests in this domain, but excluded 4 children with missing data for both WISC-WMI subtests, leaving 204 children for analyses of WISC-VCI and 200 for analyses of WISC-WMI. This approach was also used to impute data for 2 children missing the WAIS-III maternal intelligence score, which was a covariate in all models. For other covariates, missing values were replaced by the modal value: prepregnancy maternal body mass index (BMI) (< 25 kg/m^2^; *n* = 1); maternal education (> 12 years; *n* = 1); breastfeeding (> 16 weeks; *n* = 1); maternal alcohol consumption at the beginning of pregnancy (no; *n* = 4); household renovation and hobbies during childhood likely to involve exposure to organic solvents (yes; *n* = 3).

**Table 2:** Concentrations of GE metabolites in urine samples of 204 pregnant women randomly selected from the PELAGIE mother–child cohort.

Urinary metabolites	Standard for calibration	Frequency of detection (%)	Median (mg/L)	Interquartile range
Methoxyacetic acid (MAA)	Acros Organic	197 (97%)	0.062	(0.033–0.100)
Ethoxyacetic acid (EAA)	Sigma Aldrich	191 (94%)	0.016	(0.010–0.027)
Ethoxyethoxyacetic acid (EEAA)	A2S	185 (91%)	0.028	(0.010–0.079)
2-Butoxyacetic acid (BAA)	Acros Organic	203 (100%)	0.042	(0.022–0.070)
Phenoxyacetic acid (PhAA)	Dr Erhenstorfer	204 (100%)	0.390	(0.170–0.990)
The limit of detection was 0.003 mg/L for all metabolites.				

Multivariable linear regression models were constructed to explore associations between the WISC scores of 6-year-old children and the GE metabolite concentrations in maternal prenatal urine samples. Because the NEPSY scores were count variables, we used Poisson regression models to explore their associations with these concentrations.

Urinary concentrations were first categorized in three tertiles and then used continuously after natural log-transformation, in view of the log-normal distribution of the data. Associations with GE modeled as log-transformed continuous variables were not reported for exposure–outcome associations with a significant departure from linearity. The linearity of the relation between these metabolite concentrations (in natural log-scale) and the WISC scores was assessed by using restricted cubic splines based on full multivariate models (Desquilbet and Mariotti 2010). The assumption of linearity was rejected when the nonlinear part of the restricted cubic spline model significantly improved the fit of the linear model (*p* < 0.05). We chose the 25th, 50th, and 75th percentiles as knots. Because the method from Desquilbet and Mariotti (2010) was not appropriate for Poisson regression, we assessed monotonic trends with NEPSY subtests with the log-likelihood ratio test, comparing models including the metabolite concentrations in tertiles as a categorical variable to models including the concentrations in tertiles as a continuous variable. The median concentration was assigned to each tertile.

The following covariates were *a priori* included in all models: the HOME score (continuous), the WAIS score (continuous), maternal education (≤ 12 years, > 12 years), mother’s urinary creatinine concentration (continuous), and the investigator who administered the WISC and NEPSY tests. In addition, the models included covariates associated with both urinary concentrations of the GE metabolites and the corresponding neuropsychological score at *p* < 0.2, based on the relevant univariate analyses (Student tests, ANOVA or Pearson correlation coefficients for WISC domains; Wilcoxon sum rank tests, Kruskal–Wallis or Spearman correlation coefficients for GE metabolites and for NEPSY subsets). The following covariates were assessed: prepregnancy maternal BMI (≤ 25, > 25 kg/m^2^), maternal age (continuous), sex, parity (0, ≥ 1), breastfeeding (none, ≤ 16, > 16 weeks), maternal tobacco consumption at the beginning of pregnancy (yes/no), number of smokers living in the household at birth (0, 1, ≥ 2), maternal alcohol consumption at the beginning of pregnancy (yes/no), habitual fish consumption before pregnancy (≤ 1, ≥ 2 time per week), place of residence at child age 6 years (urban, rural), child’s school level at the time of the interview (nursery school, primary school), siblings at age 6 years (continuous), number of cigarettes smoked daily in the household at age 6 years (0, ≤ 10, > 10), child’s sleep duration (< 10.5, 10.5–11, > 11 hr per day), acid-leachable lead in floor dust (≤ 1, 1–3, > 3 μg/m^2^), usage of cleaning products during childhood (< 8, ≥ 8 times per month), household renovation and hobbies during childhood likely to involve exposure to organic solvents (yes/no). Finally, in WISC models, adjustments were made for breastfeeding (methoxyacetic acid; MAA), alcohol consumption (ethoxyethoxyacetic acid; EEAA), parity (BAA; 2-butoxyacetic acid), and fish consumption (BAA). In NEPSY models, additional adjustments were made for breastfeeding (MAA), number of cigarettes smoked daily in the household at age 6 (MAA, EEAA), prepregnancy maternal body mass index (ethoxyacetic acid, EAA; EEAA; BAA), maternal age (EAA), household renovation likely to involve exposure to organic solvents (EAA), alcohol consumption (EEAA), parity (BAA), and fish consumption (BAA). Considering the weak correlation between GE metabolites (see Table S2), we did not mutually adjust for exposure to multiple GE. Linearity of the relation between continuous covariates and the outcomes was assessed with the method presented above. The shape of the nonlinear relations was defined with generalized additive models. Because of their quadratic trend, the HOME scores were squared in the WISC-WMI models.

We assessed the normal distribution of residuals for the linear regression models. We also checked the influence of statistical outliers according to the studentized residuals (linear models) or standardized residual deviance (Poisson regression models), by excluding these outliers from the analyses. Outliers were defined by values below –2 or above 2. Sensitivity analyses to assess the impact of imputation on our results were restricted to participants with no missing value for covariates, WISC subtests, or NEPSY scores in any of the GE models for each association (*n* = 184 for WISC models and 194 for NEPSY models). Statistical significance was defined by a *p*-value < 0.05. All statistical analyses used SAS software V9.4 (SAS Institute Inc.).

### Ethics Statements

All adult participants provided written informed consent. Children provided verbal and witnessed assent. This study was approved by the French Consulting Committee for the Treatment of Information in Medical Research (no. 09.485) and by the French National Commission for the Confidentiality of Computerized Data (no. 909347).

## Results

### Description of the Population

Table 3 summarizes the characteristics of the 204 mother–child pairs studied. At the beginning of pregnancy, the mothers’ mean age was 30 years; most were multiparous (60%), had a prepregnancy BMI < 25 kg/m^2^ (81%), did not smoke (77%), and had completed college (67%). The children’s mean birth weight was 3,432 g, 72% were breastfed (38% for > 16 weeks), and 53% were girls. Median scores were 108 for WISC-VCI (IQR, 98–116), 104.5 for WISC-WMI (IQR, 97–118), 13 for Design Copying (IQR, 10–15), and 12 for Arrows (IQR, 11–14) (Table 4).

**Table 3:** Characteristics of the population (*n* = 204).

Characteristic	*n* (%)	Mean ± SD
Maternal factors
Maternal age at conception (years)	204 (100.0)	30.3 ± 4.2
Parity
0	82 (40.2)
≥ 1	122 (59.8)
Education
≤ 12 years	67 (32.8)
> 12 years	136 (66.7)
Missing	1 (0.5)
Body mass index (kg/m^2^)
≤ 25	164 (80.4)
> 25	39 (19.1)
Missing	1 (0.5)
Fish consumption before pregnancy
Eaten no more than once a week	144 (70.6)
Eaten more than once a week	60 (29.4)
Alcohol consumption during pregnancy
No	175 (85.8)
Yes	25 (12.2)
Missing	4 (2.0)
Tobacco consumption in the households at age 6 years
None	115 (56.4)
≤ 10 cigarettes per day	45 (22.1)
> 10 cigarettes per day	44 (21.5)
Household renovation during childhood likely to expose to organic solvents
No	68 (33.8)
Yes	133 (66.2)
WAIS score	202 (99.0)	93.6 ± 11.22
Urinary creatinine (g/L)		1.06 ± 0.46
Child factors
Sex
Boys	95 (46.6)
Girls	109 (53.4)
Birth weight (g)		3,432 ± 392
Gestational age at birth (weeks)		39.5 ± 1.2
Breastfeeding
None	57 (27.9)
≤ 16 weeks	69 (33.8)
> 16 weeks	77 (37.8)
Missing	1 (0.5)
HOME score		46.3 ± 3.8
Investigator
1	97 (47.6)
2	107 (52.4)

**Table 4:** Description of neurocognitive abilities and visuospatial skills of the 6-year-old children (*n *= 204).

Outcome	*n*	Median (25th–75th percentile)	90th percentile
Neurocognitive abilities
WISC–Verbal Comprehension Index	204	108.0 (98.0–116.0)	126.0
WISC–Working Memory Index	200	104.5 (97.0–118.0)	125.5
Visuospatial skills
NEPSY–Design Copying	204	13 (10–15)	16
NEPSY–Arrows	204	12 (11–14)	15

Table 2 presents the detection frequency and median values of the five GE metabolites measured in the prenatal urine samples. Detection frequencies ranged from 91% (for EEAA) to 100% (for phenoxyacetic acid; PhAA). Median concentrations ranged from 0.016 (for EAA) to 0.39 mg/L (for PhAA).

### GE Metabolites and WISC Scores

In general, adjustment did not substantially modify associations between the individual GE and WISC-VCI and WISC-WMI scores (Table 5). Estimated associations with WISC-WMI were generally close to the null and none were significant. Prenatal urinary concentrations of PhAA were associated with lower WISC-VCI scores, with a significant association for the third versus first tertile [β_adjusted_ = –6.53; 95% confidence interval (CI): –11.44, –1.62], and a negative but nonsignificant association for the second tertile (β_adjusted_ = –4.72; 95% CI: –9.61, 0.16). Corresponding estimates for the other GEs and WISC-VCI were closer to the null and none were significant. The restricted cubic spline model for PhAA and WISC-VCI indicated a significant departure from linearity (*p* = 0.012), with a negative slope for PhAA urine concentrations below the median value (0.39 mg/L) followed by a plateau above the median value (see Figure S1). Otherwise, there were no significant departures from linearity for associations between GE and WISC scores.

**Table 5:** Associations between concentrations of glycol ether metabolite in mothers’ prenatal urine samples and the WISC-IV scores of their 6-year-old children.

Metabolite (mg/L)	*n*	WISC-Verbal Comprehension Index		*n*	WISC-Working Memory Index	
		β_crude_ (95% CI)	β_adjusted_ (95% CI)		β_crude_ (95% CI)	β_adjusted_ (95% CI)
MAA
≤ 0.043	68	Reference	Reference	67	Reference	Reference
> 0.043–0.081	67	0.59 (–4.74, 5.93)	1.75 (–3.15, 6.65)	65	–0.04 (–5.03, 4.94)	0.40 (–4.35, 5.15)
> 0.081	69	4.06 (–1.24, 9.36)	2.87 (–2.00, 7.74)	68	0.55 (–4.38, 5.48)	–0.33 (–5.03, 4.37)
Continuous (log)	204	1.39 (–0.51, 3.30)	0.95 (–0.83, 2.73)	200	1.16 (–0.64, 2.96)	1.00 (–0.74, 2.74)
EAA
≤ 0.011	68	Reference	Reference	67	Reference	Reference
> 0.011–0.021	67	2.73 (–2.63, 8.09)	3.35 (–1.66, 8.36)	65	0.34 (–4.65, 5.32)	0.09 (–4.70, 4.88)
> 0.021	69	1.42 (–3.90, 6.74)	–1.40 (–6.58, 3.78)	68	–0.69 (–5.62, 4.24)	–3.16 (–8.13, 1.80)
Continuous (log)	204	0.29 (–1.96, 2.55)	–0.29 (–2.48, 1.89)	200	0.33 (–1.78, 2.44)	–0.25 (–2.35, 1.86)
EEAA
≤ 0.016	68	Reference	Reference	67	Reference	Reference
> 0.016–0.050	66	4.97 (–0.36, 10.31)	4.51 (–0.39, 9.40)	65	1.32 (–3.66, 6.30)	0.41 (–4.31, 5.13)
> 0.050	70	0.44 (–4.82, 5.70)	–0.22 (–5.30, 4.86)	68	1.31 (–3.62, 6.24)	0.16 (–4.78, 5.09)
Continuous (log)	204	0.53 (–0.62, 1.69)	0.31 (–0.79, 1.42)	200	0.47 (–0.63, 1.57)	0.21 (–0.87, 1.29)
BAA
≤ 0.028	67	Reference	Reference	66	Reference	Reference
> 0.028–0.058	68	–1.14 (–6.50, 4.23)	–0.58 (–5.55, 4.40)	67	–1.09 (–6.06, 3.87)	–1.57 (–6.39, 3.26)
> 0.058	69	–1.91 (–7.26, 3.43)	–2.25 (–7.22, 2.72)	67	–0.27 (–5.24, 4.69)	–1.49 (–6.32, 3.34)
Continuous (log)	204	–0.22 (–2.59, 2.15)	–0.61 (–2.84, 1.61)	200	1.04 (–1.15, 3.22)	0.45 (–1.71, 2.60)
PhAA
≤ 0.224	68	Reference	Reference	68	Reference	Reference
> 0.224–0.781	67	–5.16 (–10.44, 0.12)	–4.72 (–9.61, 0.16)	65	–0.17 (–5.14, 4.80)	0.41 (–4.34, 5.16)
> 0.781	69	–6.61 (–11.89, –1.37)	–6.53 (–11.44, –1.62)	67	0.35 (–4.48, 5.28)	0.93 (–3.85, 5.70)
Continuous (log)	204	NA	NA	200	0.24 (–0.85, 1.32)	0.08 (–0.97, 1.14)
Abbreviations: BAA, 2-butoxyacetic acid; EAA, ethoxyacetic acid; EEAA, ethoxyethoxyacetic acid; MAA, methoxyacetic acid; NA: Not applicable, significant departure from linearity based on restricted cubic spline model; PhAA, phenoxyacetic acid. All adjusted models were adjusted for education, WAIS score, HOME score, creatinine concentration, and investigator who administered the neurocognitive test. Additional adjustments were made for breastfeeding (MAA), alcohol consumption (EEAA), parity (BAA), and fish consumption (BAA). WISC-IV scores are based on a scale with a mean of 100 and a standard deviation of 15 (minimum–maximum, 40–160).						

These conclusions did not change in sensitivity analyses restricted to participants with complete data (*n* = 184; see Table S3). After exclusion of 13 statistical outliers, the association between the WISC-VCI score and the maternal urinary concentrations of PhAA remained statistically significant (third vs. first tertile: β_adjusted_ = –5.29; 95% CI: –9.64, –0.94; second vs. first tertile: β_adjusted_ = –4.47; 95% CI: –8.76, –0.17), and its shape remained similar (see Table S4 and Figure S2). Data were not shown for other associations.

### GE Metabolites and NEPSY Scores

Table 6 reports the associations between urinary concentrations of GE metabolites and the NEPSY visuospatial domain. There were no significant departures from linearity based on our methodology (data not shown). The crude analyses showed no associations. After adjustment, the highest prenatal urinary concentrations of EAA were associated with a lower score on the Design Copying NEPSY subtest (third vs. first tertile: β_adjusted_ = –0.11; 95% CI: –0.21, 0.00; second vs. first tertile: β_adjusted_ = 0.03; 95% CI: –0.07, 0.13; using continuous log-scale variable: β_adjusted_ = –0.01; 95% CI: –0.06, 0.03).

**Table 6:** Associations between concentrations of glycol ether metabolite in mothers’ prenatal urine samples and the visuospatial skills (NEPSY) of their 6-year-old children.

Metabolite (mg/L)	*n*	NEPSY–Design Copying		*n*	NEPSY–Arrows	
		β_crude_ (95% CI)	β_adjusted_ (95% CI)		β_crude_ (95% CI)	β_adjusted_ (95% CI)
MAA
≤ 0.043	68	Reference	Reference	68	Reference	Reference
> 0.043–0.081	67	0.02 (–0.08, 0.11)	0.02 (–0.08, 0.12)	67	0.02 (–0.08, 0.11)	0.02 (–0.08, 0.12)
> 0.081	69	0.05 (–0.05, 0.14)	0.04 (–0.06, 0.13)	69	0.03 (–0.07, 0.12)	0.01 (–0.08, 0.11)
Continuous (log)	204	0.02 (–0.01, 0.06)	0.02 (–0.02, 0.06)	204	0.01 (–0.03, 0.04)	0.01 (–0.03, 0.04)
EAA
≤ 0.011	68	Reference	Reference	68	Reference	Reference
> 0.011–0.021	67	0.04 (–0.05, 0.14)	0.03 (–0.07, 0.13)	67	0.01 (–0.09, 0.10)	0.01 (–0.10, 0.11)
> 0.021	69	–0.05 (–0.14, 0.05)	–0.11 (–0.21, 0.00)	69	0.01 (–0.09, 0.10)	–0.02 (–0.12, 0.09)
Continuous (log)	204	0.00 (–0.04, 0.04)	–0.01 (–0.06, 0.03)	204	0.01 (–0.03, 0.05)	0.00 (–0.04, 0.05)
EEAA
≤ 0.016	68	Reference	Reference	68	Reference	Reference
> 0.016–0.050	66	–0.04 (–0.14, 0.05)	–0.06 (–0.16, 0.04)	66	–0.07 (–0.17, 0.03)	–0.08 (–0.18, 0.02)
> 0.050	70	–0.07 (–0.16, 0.03)	–0.10 (–0.20, 0.01)	70	0.01 (–0.09, 0.10)	0.00 (–0.11, 0.10)
Continuous (log)	204	0.00 (–0.03, 0.02)	–0.01 (–0.03, 0.01)	204	0.01 (–0.01, 0.03)	0.01 (–0.01, 0.03)
BAA
≤ 0.028	67	Reference	Reference	67	Reference	Reference
> 0.028–0.058	68	–0.01 (–0.10, 0.09)	–0.01 (–0.11, 0.09)	68	0.00 (–0.09, 0.10)	0.00 (–0.10, 0.10)
> 0.058	69	–0.02 (–0.11, 0.08)	–0.03 (–0.13, 0.06)	69	–0.03 (–0.13, 0.06)	–0.05 (–0.15, 0.05)
Continuous (log)	204	0.02 (–0.02, 0.06)	0.01 (–0.04, 0.05)	204	0.00 (–0.04, 0.05)	–0.01 (–0.05, 0.04)
PhAA
≤ 0.224	68	Reference	Reference	68	Reference	Reference
> 0.224–0.781	67	0.00 (–0.10, 0.10)	0.00 (–0.09, 0.10)	67	0.05 (–0.05, 0.14)	0.05 (–0.05, 0.15)
> 0.781	69	–0.01 (–0.11, 0.08)	–0.01 (–0.11, 0.08)	69	0.03 (–0.07, 0.13)	0.04 (–0.06, 0.14)
Continuous (log)	204	0.00 (–0.02, 0.02)	–0.01 (–0.03, 0.02)	204	0.01 (–0.01, 0.03)	0.01 (–0.02, 0.03)
Abbreviations: BAA, 2-butoxyacetic acid; EAA, ethoxyacetic acid; EEAA, ethoxyethoxyacetic acid; MAA, methoxyacetic acid; PhAA, phenoxyacetic acid. All adjusted models were adjusted for education, WAIS score, HOME score, creatinine concentration, and investigator who administered the neurocognitive test. Additional adjustments were made for breastfeeding (MAA), number of cigarettes smoked per day in the household at age 6 (MAA, EEAA), prepregnancy maternal body mass index (EAA, EEAA, BAA), maternal age (EAA), household renovation likely to involve exposure to organic solvents (EAA), alcohol consumption (EEAA), parity (BAA,) and fish consumption (BAA). For each NEPSY subtest, the mean scaled score was 10 and the standard deviation was 3 (minimum–maximum, 1–19).						

We observed no statistical outliers in examining the association between the Design Copying subtest and the prenatal urinary concentration of EAA. When the analysis was restricted to 194 participants with complete data, the association was similar in magnitude, but no longer statistically significant (β_adjusted_ = –0.10; 95% CI: –0.21, 0.00) (see Table S5).

## Discussion

Our results show that higher urinary concentrations of PhAA during pregnancy were associated with lower scores for the WISC verbal comprehension domain in 6-year-old children, after adjustment for various potential confounders. This association was robust after excluding statistical outliers and in sensitivity analyses. The WISC-VCI reflects the ability to think, understand, conceptualize, and categorize (Wechsler 2003). The WISC-WMI, reflecting the ability to focus and to handle information, was not associated with any prenatal GE exposures studied here. Higher concentrations of EAA were associated with poorer performance in the visuospatial domain, as assessed with the Design Copying subtest of the NEPSY battery.

The high frequency of detection of these urinary GE metabolites, ranging from 91% to 100%, and their relatively short half-life in the body—from 6 to 80 hr (AFSSET 2008)—suggest that exposures were both common and repeated. This frequency of detection was higher in our study than in the few previous European studies focusing on GE exposure in the human population. Ben-Brik et al. (2004) reported detection rates ranging from 9.2% (EAA) to 67.9% (BAA) in French employed men, Fromme et al. (2013) from 45% (EAA) to 100% (PhAA) in the German general population, and Garlantézec et al. (2012) from 5.3% (EAA) to 92.9% (PhAA) in a previous analysis based on the women from the PELAGIE cohort. These differences were probably attributable to the lower detection limit in our study [0.003 mg/L vs. 0.05 mg/L for Ben-Brik et al. (2004) and Garlantézec et al. (2012)]. Median concentrations in the present study ranging from 0.016 mg/L (EAA) to 0.39 mg/L (PhAA) were close than those in other general population studies [in Fromme et al. (2013): median values ranging from < 0.01 mg/L (EAA) to 0.80 mg/L (PhAA); in Garlantézec et al. (2012): geometric means ranging from 0.07 mg/L (EAA) to 0.49 mg/L (PhAA)].

In our population, PhAA was detected at a higher median concentration (0.39 mg/L) compared with the other GE metabolites measured in the participants’ early-pregnancy urine samples (0.016–0.062 mg/L), as observed in the previous studies (Fromme et al. 2013; Garlantézec et al. 2012). PhAA is the primary metabolite of 2-phenoxyethanol (EGPhE), used as a preservative in at least half of perfumes, creams, lotions, makeup, and hair products (except dyes) on the market in France, at concentrations up to 1%. We should note that only three women in our study population worked as beauticians or hairdressers. EGPhE can also be found in pharmaceutical products and biocides, but to a much lower frequency (AFSSET 2008).

The relation between urinary concentrations of PhAA and the WISC-VCI scores appeared to be nonlinear. We observed an inverse correlation between PhAA and WISC-VCI when we considered subjects with urinary PhAA concentration below the median. Above the median, we cannot exclude the possibility of a saturation effect, a lack of sensitivity of the WISC-IV test, or residual confounding by unmeasured factors. The interpretation of this nonlinear inverse relation remains unclear.

EAA was detected at the lowest median concentration in urine samples. At the time of the urine sampling, its two most commonly used precursors were 2-(2-ethoxyethoxy)ethanol (DEGEE) and 2-[2-(2-ethoxyethoxy)ethoxy]ethanol (TEGEE). DEGEE was one of the principal GE used in cleaning products (concentration in products for domestic usage, 0.01–3%) as well as in paints, inks, biocides, and medicine in France. TEGEE was mainly used in paints (AFSSET 2008).

Only a few studies have presented evidence about neurotoxicity specifically related to GE. Morton (1990) reported persistent neurocognitive impairment in a case series of three women after 1 or 2 years of occupational exposure to EGPhE; and Musshoff et al. (1999) showed, based on *in vitro* models (*Xenopus* oocytes after injection of rat brain mRNA), that EGPhE could induce a dose-dependent attenuation of N-methyl-d-aspartate (NMDA)–induced membrane currents. NMDA receptors are crucial to the synaptic plasticity and memory and learning processes related to the hippocampus (Shapiro 2001), and hippocampal volume was correlated with the WISC–IV Full-Scale IQ score (*r* = 0.54, *p* < 0.05), and in particular with the WISC-VCI (*r* = 0.69, *p* < 0.01), in 22 children 8–18 years old (Schumann et al. 2007). Pomierny et al. (2014) suggested that EGPhE can induce oxidative stress and enhance lipid peroxidation in the frontal cortex and hippocampus of rats, but these findings might not be a specific sign of neurotoxicity. In addition, rats were exposed subcutaneously at doses described as higher than the exposure levels experienced by humans, either in occupational settings or environmentally (Pomierny et al. 2013). Other animal studies have reported behavioral impairment and variations in the concentrations of four neurotransmitters (acetylcholine, dopamine, norepinephrine, and 5-hydroxytryptamine) in the brains of young rats prenatally exposed to EGEE, a parent compound of EAA (Nelson and Brightwell 1984; Nelson et al. 1984). In these two studies, rats were exposed by inhalation at a concentration of 100 ppm, which corresponded to the half of the permissible exposure limit applicable at the time of the study. The study by Nelson et al. (1984) also suggested behavioral impairment in rats prenatally exposed to EGME (parent compound of MAA), but we found no association with MAA in our results. Pomierny et al. (2013) found a decreased total antioxidant response, increased lipid peroxidation, and increased caspase-3 activity in the frontal cortex and the hippocampus of adult rats exposed to a mixture of EGBE (parent compound of BAA) and EGME or EGEE. However, as said above, these effects might be nonspecific and cannot be directly linked to a toxic effect on neurodevelopment. To our knowledge, no study has yet focused on the possible neurotoxicity of EEAA. Overall, these findings tend to support the plausibility of our results about PhAA and EAA and reinforce the need for further studies about the neurotoxic potential of GE.

The inverse association between EAA and Design Copying observed in the present study is consistent with previous findings by Till et al. (2001a). Based on a prospective cohort design, they suggested lower graphomotor ability (a composite score based on Design Copying and Visuo-Motor Precision subtests of the NEPSY battery) in 3- to 7-year-old children of women occupationally exposed to organic solvents (*n* = 33), compared with children of unexposed women (*n* = 27). Till et al. (2001a) also found that poorer performance in receptive language abilities (Phonological Processing, Comprehension of Instructions, and Peabody Picture Vocabulary Test) was associated with prenatal exposure to organic solvents; but these subtests were not assessed in our study.

On the other hand, two studies that assessed the impact of organic solvents on children’s verbal abilities did not report poorer performance (Eskenazi et al. 1988; Laslo-Baker et al. 2004), but their sample sizes were 82 and 64 children, respectively. Conversely, a large Dutch study suggested that children of women working as hairdressers (*n* = 9,000) spoke later than children of clothing saleswomen (*n* = 9,000) (Kersemaekers et al. 1997). Overall, these studies assessed only occupational exposures, and none explored GE specifically. In addition, concerning the latter study, the exposure of hairdressers to a wide range of chemicals makes comparison with our results difficult.

The PELAGIE mother–child cohort was designed to explore the impact of prenatal chemical exposure on childhood development (Garlantézec et al. 2009; Pelé et al. 2013; Petit et al. 2012). The collection of a large set of covariates makes it possible to adjust statistical analyses for various lifestyle factors, domestic habits and exposures, indicators including the mother’s educational level and verbal intelligence quotient (WAIS score), and the stimulation of the child’s home environment (HOME score).

We used first morning void urine samples collected during the first trimester to estimate prenatal exposures to GE from all sources. In view of the fast clearance of GE metabolites from the body and the possible day-to-day variation in exposure intensity, it is likely that our single spot urine measurement did not capture intraindividual variability over time. First morning void urine samples were likely done before the morning cosmetic application and would thus reflect only a part of the previous day’s exposure. In addition, although creatinine correction is commonly used to adjust for urine dilution when examining urinary biomarkers, pregnancy causes variations in creatinine metabolism and excretion, which may have introduced errors in considering urine dilution. Considering the multiple comparisons performed and the possible measurement errors, we cannot rule out the possibility of chance findings regarding the associations observed with PhAA and EAA. Nevertheless, these associations are consistent with existing animal data and robust to the exclusion of statistical outliers. Although the collection and analysis of repeated samples would probably have enhanced the precision of the exposure estimation in our situation, adequate precision would have required a substantial number of samples (Perrier et al. 2016). Finally, we cannot rule out the possibility that these results may be explained by one of the various chemicals used in combination with GE. In 2012, the French Drug and Cosmetic Safety agency (ANSM 2012) studied 43 cosmetic products containing 2-phenoxyethanol and found that the chemical family of preservative most frequently associated with EGPhE was parabens (methyl-, ethyl-, propyl-, or/and butyl-paraben) in 33 of these 43 products. Sodium benzoate and methyldibromoglutaronitrile were also associated with it, but to a lesser extent. To our knowledge, associations between these chemicals and neurodevelopmental outcomes have not been evaluated in human studies.

## Conclusion

To our knowledge, this is the first study to explore the impact of prenatal exposure to five GE metabolites on children’s neurodevelopment. Higher urinary concentrations of PhAA during the first trimester of pregnancy were associated with lower WISC-IV verbal comprehension domain scores in our study population of 6-year-old children. In addition, higher prenatal urine concentrations of EAA were associated with poorer visuospatial performance based on the NEPSY Design Copying subtest. Precursors of PhAA and EAA are GE currently used in Europe in cosmetics and cleaning agents. Because the present study is of modest sample size and that uncontrolled confounding cannot be ruled out, these findings need to be replicated on a larger population. Additional studies are also needed to improve knowledge about the sources of GE exposure, the toxicokinetics of these compounds and their metabolites, and the underlying biological mechanisms that might explain these associations.

## Supplemental Material

(458 KB) PDF
