# Supplementary material for: Prenatal Exposure to Glycol Ethers and Neurocognitive Abilities in 6-Year-Old Children: The PELAGIE Cohort Study
Source: Environ Health Perspect. 2016 Oct 14;125(4):684–90. doi: 10.1289/EHP39 (PMC5381990; doi:10.1289/EHP39)
Supplement: (458 KB) PDF [file EHP39.s001.acco.pdf]

Environ Health Perspect
DOI: 10.1289/EHP39

Note to readers with disabilities: *EHP* strives to ensure that all journal content is accessible to all readers. However, some figures and Supplemental Material published in *EHP* articles may not conform to 508 standards due to the complexity of the information being presented. If you need assistance accessing journal content, please contact ehponline@niehs.nih.gov. Our staff will work with you to assess and meet your accessibility needs within 3 working days.

Supplemental Material

Prenatal Exposure to Glycol Ethers and Neurocognitive Abilities in 6-Year-Old Children: The PELAGIE Cohort Study

Rémi Béranger, Ronan Garlantézec, Gaïd Le Maner-Idrissi, Agnès Lacroix, Florence Rouget, Jessica Trowbridge, Charline Warembourg, Christine Monfort, Florent Le Gléau, Marylène Jourdin, Luc Multigner, Sylvaine Cordier, and Cécile Chevrier

Table of Contents

Table S1. Characteristics of the study population compared to the rest of the cohort

Figure S1. Relation between log-transformed GE metabolite concentrations in mothers' prenatal urine samples and the WISC domains of their 6-year-old children

Table S2. Correlation between GE metabolites in mother's prenatal urine samples

Table S3. Associations between glycol ether metabolites and the WISC-IV scores of 6-year-old children in participants with no missing value for covariates (sensitivity analysis)

Table S4. Associations between PhAA concentrations in mothers' prenatal urine samples and the WISC-VCI domain of their 6-year-old children, after exclusion of statistical outliers (sensitivity analysis)

Figure S2. Relation between log-transformed PhAA concentrations in mothers' prenatal urine samples and the WISC-VCI domain of their 6-year-old children, after exclusion of statistical outliers (sensitivity analysis)

Table S5. Associations between concentrations of glycol ether metabolites and visuospatial skills (NEPSY) of 6-year-old children in participants with no missing values for covariates (sensitivity analysis)

Table S1. Characteristics of the study population compared to the rest of the cohort

Characteristics at inclusion	Study population (n=204)	Other subjects initially included in the PELAGIE cohort (n=3256)	
	n (%) or mean ± standard deviation	n (%) or mean ± standard deviation	p-value
**Maternal age at conception (year)**	30.3 ± 4.2	30.1 ± 4.3	0.39
**parity**			0.19
0	82 (40.2)	1458 (44.9)	
≥1	122 (59.8)	1786 (55.1)	
**Education**			0.14
<12 years	28 (13.8)	628 (19.3)	
=12 years	39 (19.2)	599 (18.4)	
>12 years	136 (67.0)	2158 (62.2)	
**Body mass index (kg.m^{-2})**			0.55
**≤25**	165 (80.9)	2702 (83.0)	
**>25**	39 (19.1)	554 (17.0)	
**Alcohol consumption during pregnancy**			0.31
No	175 (87.5)	2730 (84.9)	
Yes	25 (12.5)	487 (15.1)	
**Tobacco consumption at beginning of the pregnancy**			0.17
None	153 (76.1)	2312 (71.8)	
≥10 cig/day	36 (17.9)	593 (18.4)	
<10cig/day	12 (6.0)	317 (9.9)	

Comparisons performed using chi² and Student tests.

	WISC – Verbal Comprehension Index	WISC – Working Memory Index
MAA	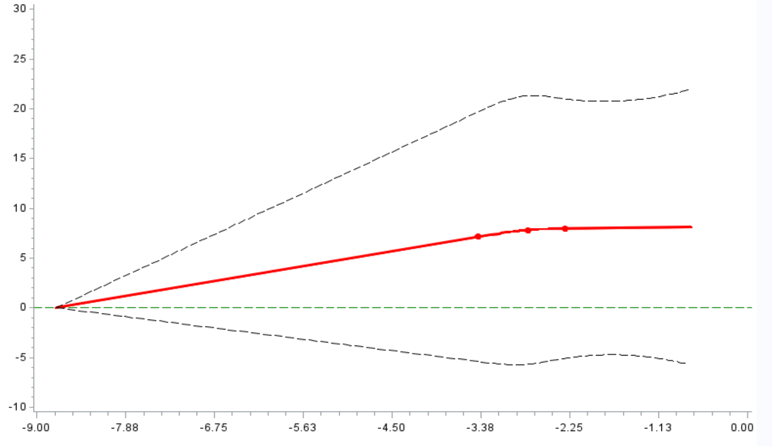<br><br>Nonlinear association: p = 0.24	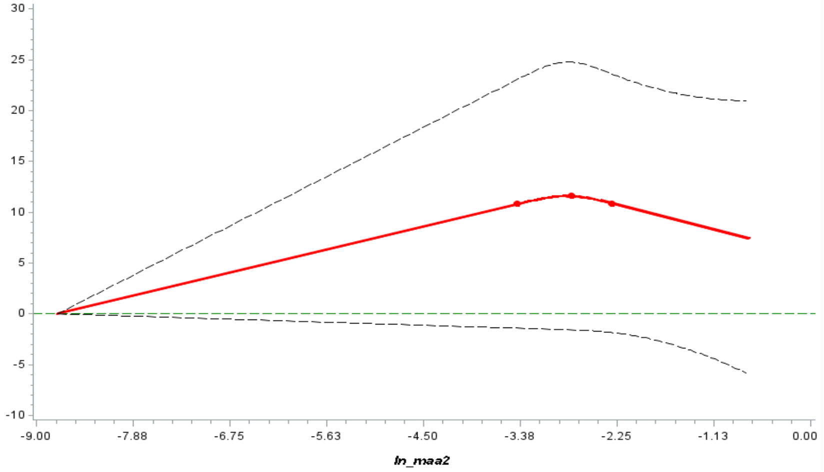<br><br>Nonlinear association: p = 0.89
EAA	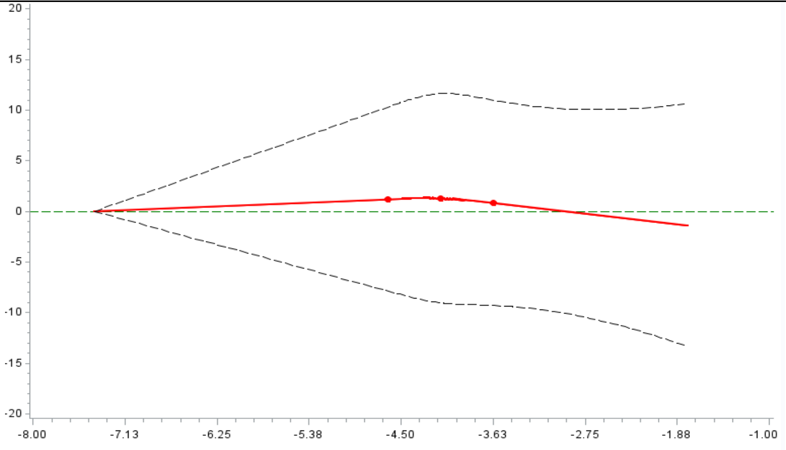<br><br>Nonlinear association: p = 0.60	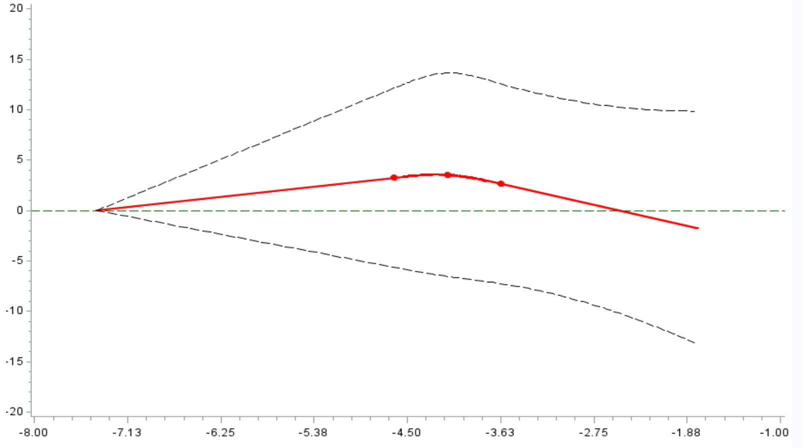<br><br>Nonlinear association: p = 0.21

EEAA	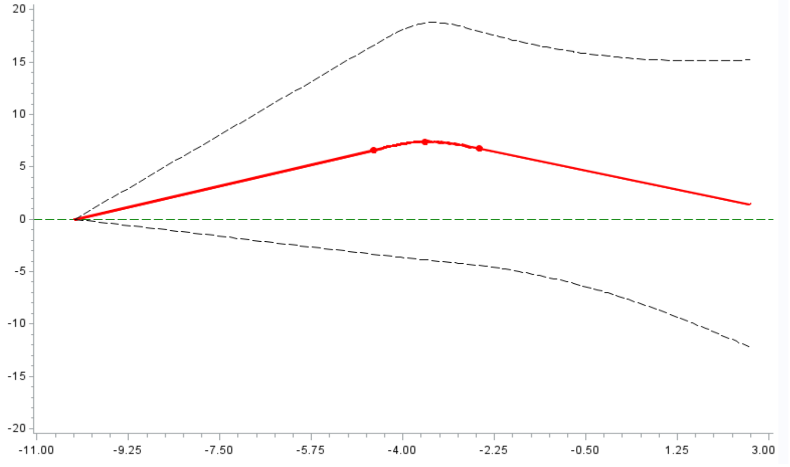<br>Nonlinear association: p = 0.98	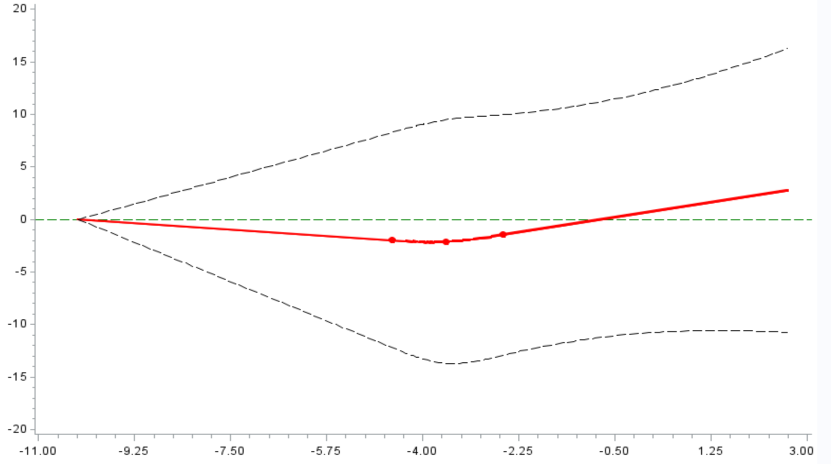<br>Nonlinear association: p = 0.95
BAA	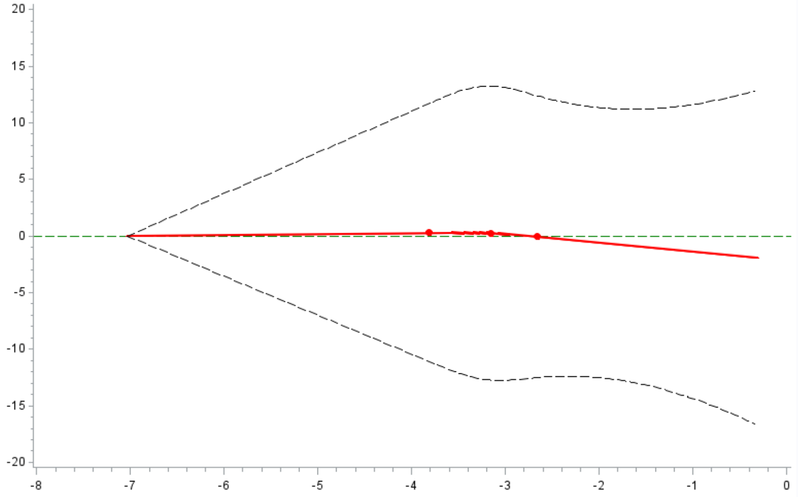<br>Nonlinear association: p = 0.39	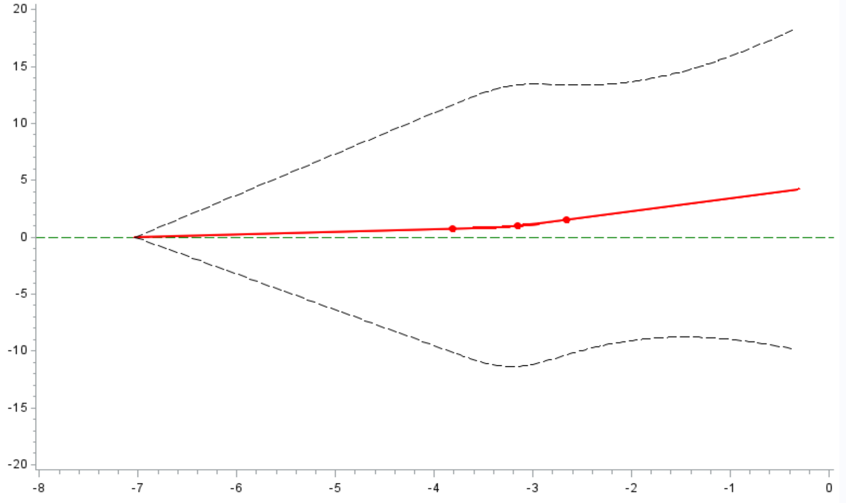<br>Nonlinear association: p = 0.51

PhAA

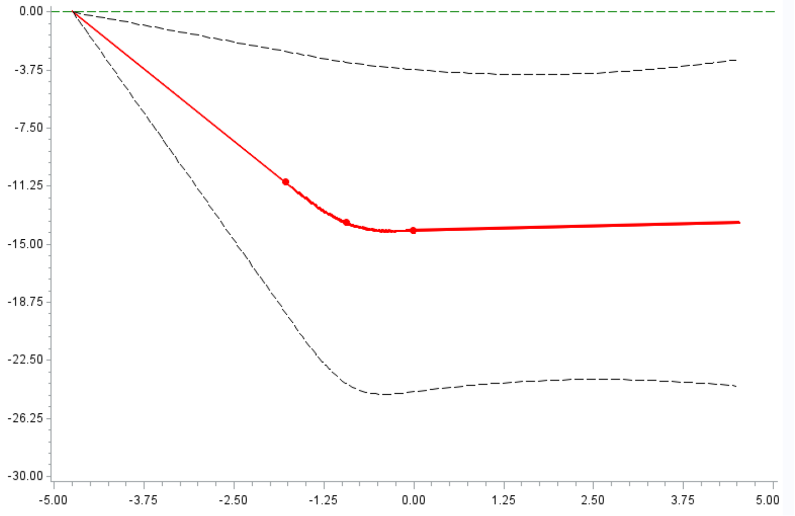

Nonlinear association: **p = 0.012**

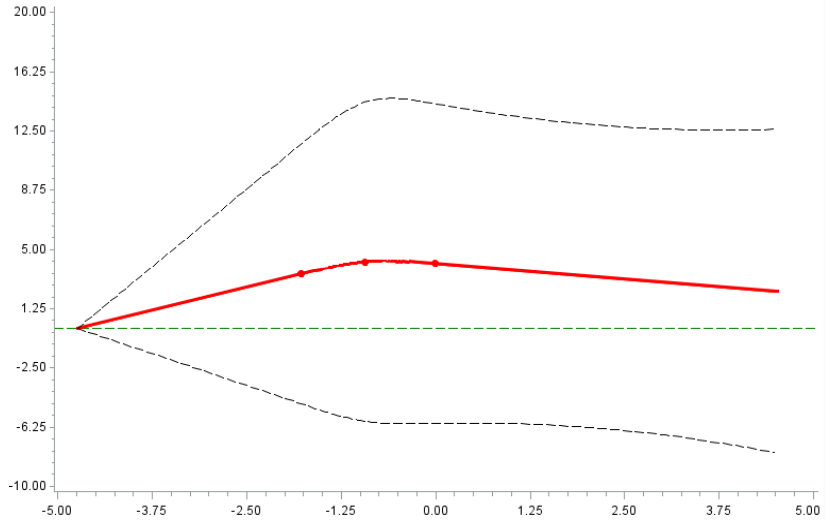

Nonlinear association: p = 0.70

Adjusted restricted cubic spline (red, continuous line) adapted to linear model and 95% confidence interval (dotted black lines). The minimum detected urinary concentration was considered as the reference value for each model. We chose the 25th, 50th, and 75th percentiles as knots. Y-axis represents the change in WISC score. X-axis represents the log of the GE metabolite urinary concentration. Abbreviation: MAA, methoxyacetic acid; EAA, ethoxyacetic acid; EEAA, ethoxyethoxyacetic acid; BAA, 2-butoxyacetic acid; PhAA, phenoxyacetic acid. All adjusted models were adjusted for education, WAIS score, HOME score, creatinine concentration, and investigators. Additional adjustments were made for breastfeeding (MAA), alcohol consumption (EEAA), parity (BAA) and fish consumption (BAA). The assumption of linearity was rejected when the non-linear part of the restricted cubic spline model was statistically significant ($p<0.05$). WISC-IV scores are based on a scale with a mean of 100 and a standard deviation of 15 (min-max: 40-160).

Figure S1. Relation between log-transformed GE metabolite concentrations in mothers' prenatal urine samples and the WISC domains of their 6-year-old children

Table S2. Correlation between GE metabolites in mother's prenatal urine samples

		MAA	EAA	E2AA	BAA	PHAA
**MAA**	rho		0,39	0,12	0,23	-0,02
	p-value		<0,001	0,09	0,001	0,78
**EAA**	rho	0,39		0,20	0,33	0,11
	p-value	<0,001		0,003	<0,001	0,10
**E2AA**	rho	0,12	0,20		0,13	0,35
	p-value	0,09	0,003		0,07	<0,001
**BAA**	rho	0,23	0,33	0,13		0,08
	p-value	0,001	<0,001	0,07		0,26
**PHAA**	rho	-0,02	0,11	0,35	0,08	
	p-value	0,78	0,10	<0,001	0,26	

Abbreviation: MAA, methoxyacetic acid; EAA, ethoxyacetic acid; EEAA, ethoxyethoxyacetic acid; BAA, 2-butoxyacetic acid; PhAA, phenoxyacetic acid. Correlation coefficient and p-value were estimated based on the Pearson correlation test. GE metabolites concentrations were log-transformed.

Table S3. Associations between glycol ether metabolites and the WISC-IV scores of 6-year-old children in participants with no missing values for covariates and outcome (sensitivity analysis)

**Metabolite**: mg/L	N	**WISC – Verbal Comprehension Index** $\beta_{adjusted}$ (95% CI)	N	**WISC – Working Memory Index** $\beta_{adjusted}$ (95% CI)
**MAA**				
≤0.043	62	ref	62	ref
>0.043 – 0.081	60	1.21 (-4.04, 6.47)	58	0.16 (-4.71, 5.03)
>0.081	62	3.44 (-1.80, 8.67)	61	-1.08 (-5.92, 3.75)
Continuous (log)	184	1.03 (-0.88, 2.95)	181	0.69 (-1.08, 2.47)
**EAA**				
≤0.011	63	ref	63	ref
>0.011-0.021	56	5.27 (-0.15, 10.70)	54	2.20 (-2.77, 7.18)
>0.021	65	-0.37 (-5.83, 5.09)	64	-2.54 (-7.57, 2.49)
Continuous (log)	184	0.04 (-2.29, 2.37)	181	-0.01 (-2.13, 2.12)
**EEAA**				
≤0.016	57	ref	57	ref
>0.016-0.050	60	3.03 (-2.31, 8.37)	59	0.94 (-3.98, 5.87)
>0.050	67	-1.65 (-7.11, 3.81)	65	-1.03 (-6.09, 4.04)
Continuous (log)	184	-0.14 (-1.42, 1.14)	181	-0.09 (-1.26, 1.09)
**BAA**				
≤0.028	62	ref	62	ref
>0.028-0.058	58	0.56 (-4.80, 5.92)	57	0.22 (-4.75, 5.18)
>0.058	64	-1.75 (-7.01, 3.51)	62	-2.01 (-6.90, 2.87)
Continuous (log)	184	-0.18 (-2.51, 2.15)	181	0.65 (-1.50, 2.81)
**PhAA**				
≤0.224	58	ref	58	ref
>0.224-0.781	61	-6.04 (-11.33, -0.75)	60	-1.96 (-6.92, 2.99)
>0.781	65	-7.64 (-12.90, -2.38)	63	-0.57 (-5.52, 4.38)
Continuous (log)	184	NA	191	-0.36 (-1.44, 0.73)

NA: Not applicable, significant departure from linearity based on restricted cubic spline model. Abbreviation: MAA, methoxyacetic acid; EAA, ethoxyacetic acid; EEAA, ethoxyethoxyacetic acid; BAA, 2-butoxyacetic acid; PhAA, phenoxyacetic acid. All adjusted models were adjusted for education, WAIS score, HOME score, creatinine concentration, and investigators. Additional adjustments were made for breastfeeding (MAA), alcohol consumption (EEAA), parity (BAA), and fish consumption (BAA). WISC-IV scores are based on a scale with a mean of 100 and a standard deviation of 15 (min-max: 40-160). Analyses were restricted to participants with no missing value for covariates or WISC subtests in any of the GE models for each association.

Table S4. Associations between PhAA concentrations in mothers' prenatal urine samples and the WISC-VCI domain of their 6-year-old children, after exclusion of statistical outliers (sensitivity analysis)

		**WISC – Verbal Comprehension Index**
**Metabolite**: mg/L	N	$\beta_{adjusted}$ (95% CI)
**PhAA**		
≤0.224	61	ref
>0.224-0.781	64	-4.47 (-8.76, -0.17)
>0.781	66	-5.29 (-9.64, -0.94)

Abbreviation:PhAA, phenoxyacetic acid. Model was adjusted for education, WAIS score, HOME score, creatinine concentration, and investigators. Overall, 13 Statistical outliers were excluded (studentized residuals below -2 or above 2).

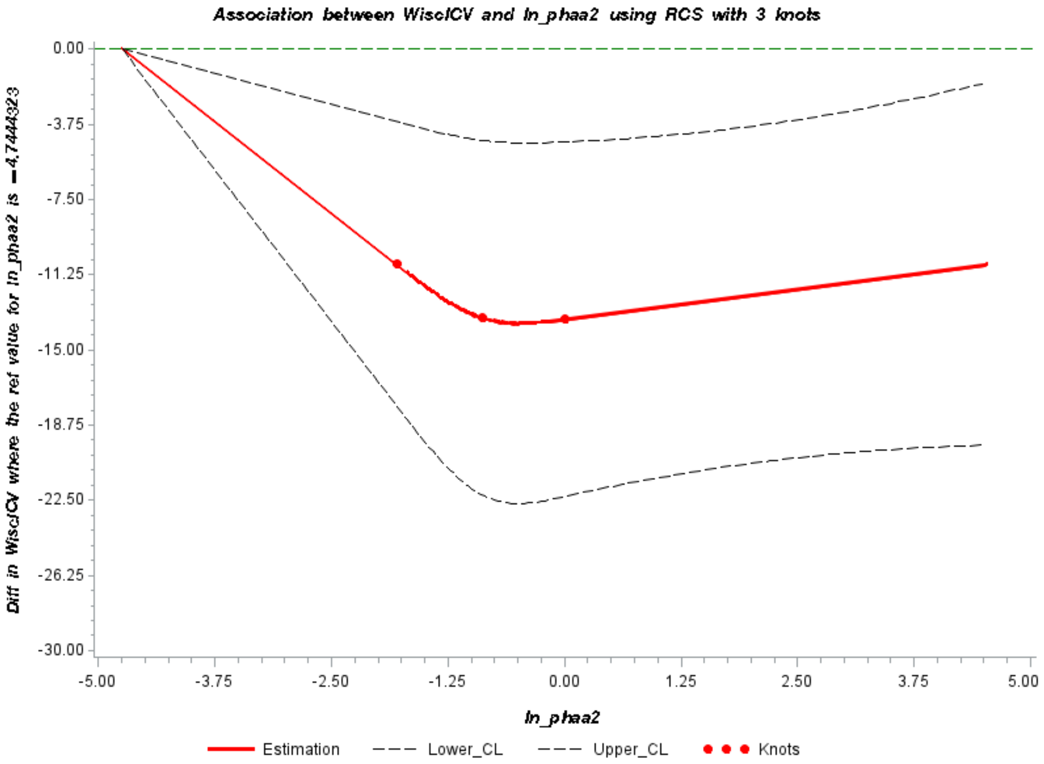

Adjusted restricted cubic spline (red, continuous line) and 95% confidence interval (dotted black lines). We chose the 25th, 50th, and 75th percentiles as knots. Y-axis represents the change in WISC score. X-axis represents the log of the GE metabolite urinary concentration. Abbreviation: PhAA, phenoxyacetic acid. Model was adjusted for education, WAIS score, HOME score, creatinine concentration, and investigators. Overall, 13 Statistical outliers were excluded (studentized residuals below -2 or above 2).

Figure S2. Relation between log-transformed PhAA concentrations in mothers' prenatal urine samples and the WISC-VCI domain of their 6-year-old children, after exclusion of statistical outliers (sensitivity analysis)

Table S5. Associations between concentrations of glycol ether metabolites and the visuospatial skills (NEPSY) of 6-year-old children in participants with no missing values for covariates and outcome (sensitivity analysis)

		NEPSY – Design Copying		NEPSY - Arrows
**Metabolite**: mg/L	N	$\beta_{adjusted}$ (95% CI)	N	$\beta_{adjusted}$ (95% CI)
**MAA**				
≤0.043	65	ref	65	ref
>0.043 – 0.081	63	0.01 (-0.09, 0.11)	63	0.01 (-0.09, 0.11)
>0.081	66	0.03 (-0.07, 0.13)	66	0.01 (-0.09, 0.11)
Continuous (log)	194	0.02 (-0.02, 0.05)	194	0.00 (-0.03, 0.04)
**EAA**				
≤0.011	65	ref	65	ref
>0.011-0.021	60	0.03 (-0.07, 0.14)	60	0.01 (-0.10, 0.11)
>0.021	69	-0.10 (-0.21, 0.00)	69	-0.01 (-0.12, 0.09)
Continuous (log)	194	-0.01 (-0.05, 0.03)	194	0.00 (-0.04, 0.05)
**EEAA**				
≤0.016	64	ref	64	ref
>0.016-0.050	62	-0.07 (-0.17, 0.03)	62	-0.08 (-0.18, 0.02)
>0.050	68	-0.10 (-0.20, 0.01)	68	0.00 (-0.11, 0.10)
Continuous (log)	194	-0.01 (-0.03, 0.01)	194	0.01 (-0.01, 0.03)
**BAA**				
≤0.028	64	ref	64	ref
>0.028-0.058	63	-0.01 (-0.11, 0.09)	63	0.01 (-0.09, 0.11)
>0.058	67	-0.03 (-0.13, 0.07)	67	-0.05 (-0.15, 0.05)
Continuous (log)	194	0.01 (-0.04, 0.05)	194	-0.00 (-0.05, 0.04)
**PhAA**				
≤0.224	64	ref	64	ref
>0.224-0.781	63	0.01 (-0.09, 0.11)	63	0.05 (-0.05, 0.16)
>0.781	67	-0.01 (-0.11, 0.09)	67	0.04 (-0.06, 0.14)
Continuous (log)	194	-0.01 (-0.03, 0.02)	194	0.01 (-0.02, 0.03)

Abbreviation: MAA, methoxyacetic acid; EAA, ethoxyacetic acid; EEAA, ethoxyethoxyacetic acid; BAA, 2-butoxyacetic acid; PhAA, phenoxyacetic acid. All adjusted models were adjusted for education, WAIS score, HOME score, creatinine concentration, and investigators. Additional adjustments were made for breastfeeding (MAA), number of cigarettes smoked per day in the household at age 6 (MAA, EEAA), prepregnancy maternal body mass index (EAA, EEAA, BAA), maternal age (EAA), household renovation likely to involve exposure to organic solvents (EAA), alcohol consumption (EEAA), parity (BAA,) and fish consumption (BAA). For each NEPSY subtest, the mean scaled score was 10 and the standard deviation was 3 (min-max: 1-19). Analyses were restricted to participants with no missing value for covariates or NEPSY scores in any of the GE models for each association.
